# Comorbidity between lung cancer and COVID-19 pneumonia: role of immunoregulatory gene transcripts in high *ACE2*-expressing normal lung

**DOI:** 10.1177/17588359221133893

**Published:** 2022-10-28

**Authors:** Vladimir Lazar, Jacques Raynaud, Shai Magidi, Catherine Bresson, Jean-François Martini, Susan Galbraith, Fanny Wunder, Amir Onn, Gerald Batist, Nicolas Girard, Ulrik Lassen, C. S. Pramesh, Amal Al-Omari, Sadakatsu Ikeda, Guy Berchem, Jean-Yves Blay, Benjamin Solomon, Enriqueta Felip, Josep Tabernero, Eitan Rubin, Thierry Philip, Angel Porgador, Ioana Berindan-Neagoe, Richard L. Schilsky, Razelle Kurzrock

**Affiliations:** Worldwide Innovative Network (WIN) Association – WIN Consortium, 9 rue Guy Moquet, Villejuif, 94800, France; Worldwide Innovative Network (WIN) Association – WIN Consortium, Villejuif, France; Worldwide Innovative Network (WIN) Association – WIN Consortium, Villejuif, France; Worldwide Innovative Network (WIN) Association – WIN Consortium, Villejuif, France; Pfizer Inc., San Diego, CA, USA; AstraZeneca, Gaithersburg, MD, USA; Worldwide Innovative Network (WIN) Association – WIN Consortium, Villejuif, France; Sheba Medical Center, Tel-Hashomer, Israel; Segal Cancer Centre, Jewish General Hospital, McGill University, Montréal, Canada; Institut Curie, Paris, France; Rigshospitalet, Copenhagen, Denmark; Tata Memorial Hospital, Tata Memorial Centre, Homi Bhabha National Institute, Mumbai, Maharashtra, India; King Hussein Cancer Center, Amman, Jordan; Tokyo Medical and Dental University, Tokyo, Japan; Centre Hospitalier Luxembourg and Luxembourg Institute of Health, Luxembourg, Luxembourg; Centre Leon Bérard, University Lyon 1, LYRICAN & NETSARC+, Lyon, France; Avera Cancer Institute, Sioux Falls, SD, USA; Vall d’Hebron Hospital Campus and Institute of Oncology, UVic-UCC, Barcelona, Spain; Sheba Medical Center, Tel-Hashomer, Israel; Faculty of Health Sciences Ben-Gurion University of the Negev, Beer-Sheeva, Israel; Institut Curie, Paris, France; Faculty of Health Sciences Ben-Gurion University of the Negev, Beer-Sheeva, Israel; Iuliu Hatieganu University of Medicine and Pharmacy, Cluj-Napoca, Romania; Worldwide Innovative Network (WIN) Association – WIN Consortium, Villejuif, France; Worldwide Innovative Network (WIN) Association – WIN Consortium, Villejuif, France

**Keywords:** *ACE2* expression, cancer, COVID-19, normal lung, transcriptomics

## Abstract

**Background:**

SARS-CoV-2 (COVID-19) elicits a T-cell antigen-mediated immune response of variable efficacy. To understand this variability, we explored transcriptomic expression of angiotensin-converting enzyme 2 (*ACE2*, the SARS-CoV-2 receptor) and of immunoregulatory genes in normal lung tissues from patients with non-small cell lung cancer (NSCLC).

**Methods:**

This study used the transcriptomic and the clinical data for NSCLC patients generated during the CHEMORES study [*n* = 123 primary resected (early-stage) NSCLC] and the WINTHER clinical trial (*n* = 32 metastatic NSCLC).

**Results:**

We identified patient subgroups with high and low *ACE2* expression (*p* = 1.55 × 10^−19^) in normal lung tissue, presumed to be at higher and lower risk, respectively, of developing severe COVID-19 should they become infected. *ACE2* transcript expression in normal lung tissues (but not in tumor tissue) of patients with NSCLC was higher in individuals with more advanced disease. High-*ACE2* expressors had significantly higher levels of CD8+ cytotoxic T lymphocytes and natural killer cells but with presumably impaired function by high Thymocyte Selection-Associated High Mobility Group Box Protein TOX (*TOX*) expression. In addition, immune checkpoint-related molecules – *PD-L1, CTLA-4, PD-1*, and *TIGIT –* are more highly expressed in normal (but not tumor) lung tissues; these molecules might dampen immune response to either viruses or cancer. Importantly, however, high inducible T-cell co-stimulator (*ICOS*), which can amplify immune and cytokine reactivity, significantly correlated with high *ACE2* expression in univariable analysis of normal lung (but not lung tumor tissue).

**Conclusions:**

We report a normal lung immune-tolerant state that may explain a potential comorbidity risk between two diseases – NSCLC and susceptibility to COVID-19 pneumonia. Further, a NSCLC patient subgroup has normal lung tissue expressing high *ACE2* and high *ICOS* transcripts, the latter potentially promoting a hyperimmune response, and possibly leading to severe COVID-19 pulmonary compromise.

## Introduction

The functional receptor for the spike glycoprotein of SARS-CoV-2 is the angiotensin-converting enzyme 2 (ACE2). Epithelial cells that express *ACE2* in normal respiratory epithelium are the main target of the virus. SARS-CoV-2 spike protein is processed by transmembrane protease-serine 2 and favors binding to ACE2.^1^ SARS-CoV-2 can enter *ACE2*-expressing cells, but not cells without ACE2.^2^ Higher levels of *ACE2* expression have been seen in lung tissues of patients with severe COVID-19.^3^ After entry into the cells, coronaviruses activate aryl hydrocarbon receptors (AhRs) by an indoleamine 2,3-dioxygenase (*IDO1*)-independent mechanism, bypassing the *IDO1*-kynurenine-AhR pathway.^4^ However, AhRs enhance their own activity through an *IDO1*-AhR-*IDO1*-positive feedback loop prolonging activation.^4^

Infection with SARS-CoV-2 induces priming of the T-cell receptor (TCR), triggering a T-cell antigen-mediated cellular (cytotoxic) and humoral (neutralizing antibodies) immune response.^5^ In most people infected with SARS-CoV-2, an adequate immune response is able to control the infection and clear the viral load, allowing recovery and development of persistent immunity. However, some individuals develop a severe form of COVID-19 (defined as hospitalization, and/or admission to the intensive care unit, and/or intubation/mechanical ventilation, and/or death) that may result from an over-reacting immune and inflammatory response, called cytokine storm.^6–9^ This hyperimmune response can trigger extensive damage to the normal lung tissues, resulting in acute respiratory compromise, multiple organ failure, and death.

Like SARS-CoV-2, cancer induces presentation of specific foreign/neo-antigens that prime TCRs and induce a T-cell antigen-mediated immune response.^10,11^ While recent observations demonstrated increased expression of immune checkpoint receptors (including *PD-1* and *CTLA-4*, both of which have been implicated in cancer development) in lung tissues of people with COVID-19,^12^ the impact of this expression pattern on the severity of COVID-19 infection remains unclear; however, it has been suggested that the deterioration of many patients with COVID-19 is driven by an immune-mediated cytokine release syndrome that theoretically could be adversely potentiated by immunotherapy (checkpoint blockade) as used for cancer.^13^ While it is widely believed that cancer patients infected with SARS-CoV-2 have increased mortality (regardless of anticancer therapy), the relationship between immunotherapy and COVID-19 mortality has produced both positive and negative (i.e., contradictory) reports.^14–19^

Here, we present, for the first time, an integrative biomarker analysis including transcriptomics of normal lung tissues from patients with non-small cell lung cancer (NSCLC) with the aim of better understanding the immune environment of lung cancer patients who might be at risk of developing severe COVID-19 illness.

## Materials and methods

In order to understand the immune environment of lung cancer patients and the risk for them to develop a severe form of the COVID-19, this study used the transcriptomic and the clinical data for NSCLC patients generated during the CHEMORES study^20^ [*n* = 123 primary resected NSCLC; 120 of whom had complete tumor, nodes, and metastases (TNM) staging data] and the WINTHER clinical trial^21^ (*n* = 32 metastatic NSCLC). Long-term post-surgery follow-up of the CHEMORES patients treated with curative-intent surgery enabled recording the disease-free survival (DFS), defined as the time to first recurrence, for all 123 patients. The patients had not been exposed to SARS-CoV-2 (COVID-19) as tissue collection predated the pandemic.

The main characteristics of the patients in the primary (CHEMORES) and the metastatic (WINTHER) NSCLC studies’ populations are shown in Supplemental Tables 1 and 2.

The dataset used in our *in silico* analysis consists of Agilent microarray data generated from tumor and analogous organ matched normal lung tissues from each patient.^20,21^ In the CHEMORES study,^20^ tumor and normal tissues were dissected from the tumor and the distant normal lung obtained from the lobe removed during curative-intent surgery (at distance >2 cm from the tumor). Within less than 30 min, fragments of normal and tumor tissues were snap frozen, then examined for histological content. The RNA used for microarray studies had a RNA Integrity Number >6. For metastatic NSCLC patients in WINTHER trial,^21^ tumor biopsies were obtained by 18 gauge trucut biopsies in interventional radiology units. The analogous normal biopsies were obtained from normal bronchial mucosa, using 2.5 mm forceps biopsies, in bronchoscopy units, under video control. Biopsies were stored in RNA later, then embedded and frozen in OCT (OCT emmbedding matrix). Histology quality control ensured a minimum of 50% tumor cells in tumor biopsies and a minimum of 30% normal epithelial cells in bronchial mucosa biopsies.

### Clustering patients into COVID-19 risk groups

#### Hypothesis and rationale of the study

In this study, we addressed the hypothesis that there would be identifiable biologic factors that co-segregated with high *ACE2* RNA expression, a functional receptor on cell surfaces through which SARS-CoV-2 enters the host cells, and hence a feature of cells vulnerable to COVID-19 infection.^22^ To this effect, we measured the level of gene expression (transcripts) in normal lung tissue of *ACE2*^2,3^ and of immunoregulatory genes that are currently druggable targets: *PD-L1*, inducible T-cell co-stimulator (*ICOS*), *CTLA-4; PD-1, TIGIT, PD-L2, IDO1*, and *OX40*.^19^ Furthermore, we also measured the level of expression of markers of infiltrating T cells: CD4 (helper T lymphocytes), CD8+ cytotoxic T lymphocytes, CD16 [natural killer (NK) cells], and FOXP3A (T-regulatory cells/T-regs) as well as expression of *TOX*, a marker of exhaustion of lymphocytes.^23^

The 123 patients with primary resected NSCLC from the CHEMORES study^20^ were ranked by the intensity of the expression of *ACE2* in the normal tissue from lowest to highest. The *k*-means clustering method used for the partition of the patients based on the *ACE2* expression into high and low groups (*k* = 2) (using the ‘stats’ package in R^24^) and resulted in 55 patients classified in the group of ‘low’ risk (defined by low expression of *ACE2*) and 68 patients in the group of ‘high’ risk (defined by high expression of *ACE2*). The same methodology of clustering into high and low risk groups was used also on the 32 metastatic NSCLC patients from the WINTHER trial.^21^ In the metastatic cohort, 20 patients were classified in the group of ACE2 low and the remaining 12 patients were classified in the group of ACE2 high in normal lung tissue.

### Assessing immunoregulatory gene expression differences between ACE2-high *versus* -low groups

Once the patients were classified based on the gene expression of *ACE2* into high and low risk for COVID-19 groups, the expression of selected immune-related genes was compared between the subjects in each group. The comparison was visualized using boxplots. Each boxplot includes the median (shown by the line that divides the box into two parts) and the interquartile box (shown by the box itself) which shows the middle half (from the lower quartile, represented by the lower limit of the box, to the upper quartile, represented by the upper limit of the box) expression values of the immune-related gene in each risk group. The points shown outside the box are expression values which are outside of the middle half and indicate the range of the expression values. Points shown far away from the others and from the box are outliers. The significance of the difference between the expression values of the immune-related gene in the high and low ACE2 groups was assessed by the *p* value which was derived using a two-sided Student’s *t* test, while the level of significance (*p* value) was adjusted using the Bonferroni correction (it was applied to the two-sided Student’s *t* test by multiplying the alpha by the number of performed statistical analyses). *p* Values < 0.05 after Bonferroni correction are significant.

These data comparisons were performed for both normal lung tissue and tumor tissue, both from patients with NSCLC.

### Clustering patients into gene expression level groups

Partition of patients into low and high immune-related gene groups (such as *CTLA-4* low and *CTLA-4* high, for instance) was performed similarly to how patients were classified into high and low *ACE2*-expressors, using the *k*-means clustering method (*k* = 2).

### Kaplan–Meier DFS probability plots

The Kaplan–Meier plots were generated using the ‘survival’ and ‘survminer’ packages in R. Different subgroups of patients were compared and correlated with DFS probability. Significance was tested (*p* values) for each comparison with a 95% confidence interval.

### Univariable and multivariable analysis for comparing ACE2 high/ACE2 low groups

The univariable and multivariable analyses, testing the association between binary variables (low and high ACE2 groups) and other explanatory variables (age, TNM stages, and immune-related gene expression), were performed based on a binomial logistic regression model (generalized linear model with binomial distribution) using the stats package in R.^24^ The level of significance was tested using the Wald test with 95% confidence interval. The odds ratios of the logistic regression model were calculated using the ‘oddsratio’ package in R.^25,26^

## Results

Patient data

Transcriptomic data from the normal resected lung of a total of 123 patients with resected NSCLC was available from the CHEMORES initiative (www.chemores.org).^20^ The median age of patients in the CHEMORES study was 63 years (range, 41–85 years); 72% (*n* = 89) were men. These patients had surgically resected disease with curative intent (though some patients were found at or immediately after surgery to have more advanced disease than anticipated presurgery). Complete TNM staging was available on 120 patients [56 with stage 1 disease and 64 with stage >1 (27, stage 2; 32, stage 3; 5, stage 4)], which is the number of patients used in analyses that included staging (Supplemental Table 1).

### *ACE2* transcript expression in normal lung tissues (but not in tumor tissue) of patients with NSCLC is higher in individuals with more advanced disease

**Figure 1.**
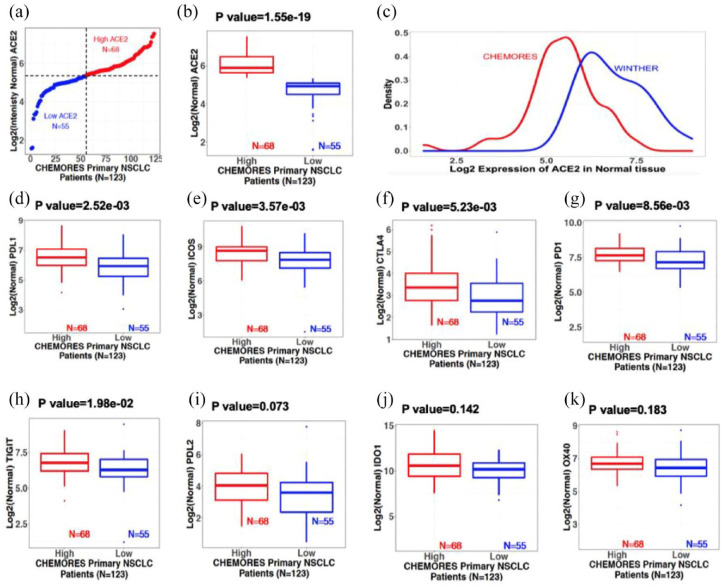
Normal lung transcriptomics from resectable NSCLC (earlier stage) and metastatic NSCLC patients. Boxplots show median and interquartile range; points outside the box, expression outliers; *p* values from two-sided Student’s *t* test (see Supplemental Figure 1 for tumor tissue). (a) Normal lung ACE2 expression in resectable NSCLC (CHEMORES dataset *n* = 123). X-axis: Patient number. Y-axis: Log 2 of normal lung tissue ACE2 expression. Patients were classified using *k*-mean clustering into high [red (*n* = 68)] and low [blue (*n* = 55)] expressors. Threshold expression (Log 2 scale) was approximately 5.35. (b) Normal lung ACE2 transcript expression boxplots from resectable NSCLC (CHEMORES dataset *n* = 123). High *versus* low ACE2 groups (*p* = 1.55 × 10^−19^). (c) Normal lung ACE2 transcript level in resectable (CHEMORES dataset *n* = 123, red) *versus* metastatic NSCLC (WINTHER dataset *n* = 32, blue). X-axis: Log 2 ACE mRNA expression. Y-axis: Expression density. Normal lung ACE2 expression in metastatic NSCLC was greater than in resectable earlier stage NSCLC. (d) to (k): Normal lung immune checkpoint expression boxplots [resectable NSCLC CHEMORES (*n* = 123)]. Patients were classified into high and low normal lung ACE2 transcript expressors (*k*-means clustering) (see Figure 1, panel A). (d) PD-L1, (e) ICOS, (f) CTLA-4, (g) PD-1, (h) TIGIT, (i) PD-L2, (j) IDO1, and (k) OX40 in each risk group. Bonferroni-adjusted *p* < 0.05 is significant. ACE2, angiotensin-converting enzyme 2; ICOS, inducible T-cell co-stimulator; NSCLC, non-small cell lung cancer.

In a cohort of 123 patients with resected NSCLC from the CHEMORES study dataset,^20^ the *ACE2* transcriptomics profile identified two distinct groups with low and high expression in normal lung tissues (collected during curative-intent surgery, at a distance >2 cm from the tumor). The threshold of *ACE2* high-low expression [Figure 1(a)] was established by *k*-means clustering method (detailed in section ‘Methods’) and identifies low- and high*-ACE2* expression groups in normal lung of our patients with NSCLC that were surgically resected (*p* = 1.55 × 10^−19^) [Figure 1(b)], outlining the significant variations of *ACE2* expression levels (low *versus* high) between individuals, which theoretically may be used to identify patients at low or high risk of developing a severe form of COVID-19.

*ACE2* transcript expression was higher in patients with more advanced TNM staging in the resectable CHEMORES dataset (Table 1).^24–26^

**Table 1.** Clinical and immunoregulatory transcriptomic expression and markers of T-cell infiltration correlate with ACE2 expression in normal lung but not in lung tumors in 120 patients with resectable NSCLC.^20,a^

	ACE2 high^b^ ACE2 low^b^ Odds ratio (95% CI) (normal lung tissue)	Univariable *p* value^f^	Multivariable *p* value
Normal lung			
Age >62.97 (*n* = 60) *versus* <62.97 years (*n* = 60)	1.609 (0.781–3.35)	0.1993	–
TNM stages >1 (*n* = 64) *versus* 1 (*n* = 56)^d^	1.79 (0.867–3.74)	0.048	0.04
PD-L1 high *versus* low^e^	2.668 (1.277–5.692)	0.00983	0.67
ICOS high *versus* low^e^	4.148 (1.945–9.143)	0.0003	0.08
CTLA-4 high *versus* low^e^	2.492 (1.197–5.308)	0.0159	0.15
PD-1 high *versus* low^e^	2.696 (1.292–5.744)	0.00895	0.69
TIGIT high *versus* low^e^	2.915 (1.395–6.237)	0.00497	0.76
CD4 high *versus* low	0.637 (0.304–1.316)	0.226	–
CD8A high *versus* low	4.853 (2.256–10.836)	0.000075	0.06
FOXP3 high *versus* low	0.702 (0.326–1.508)	0.364	–
CD16 high *versus* low	3.133 (1.426–7.112)	0.00512	0.40
TOX high *versus* low	5.259 (2.429–11.858)	0.000038	0.02
Tumor tissue			
	ACE2 high^b^ ACE2 low^b^ Odds ratioc (95% CI) (Lung tumor tissue)	Univariable *p* value^f^	Multivariable *p* value
Age ⩾62.97 (*n* = 60) *versus* <62.97 years (*n* = 60)	0.875 (0.425–1.794)	0.714	–
TNM stages >1 (*n* = 64) *versus* 1 (*n* = 56)^d^	1.018 (0.495–2.095)	0.961	–
PD-L1 high *versus* low^e^	1.154 (0.563–2.375)	0.696	–
ICOS high *versus* low^e^	1.174 (0.521–2.682)	0.699	–
CTLA-4 high *versus* low^e^	1.503 (0.671–3.458)	0.327	–
PD-1 high *versus* low^e^	1.332 (0.634–2.83)	0.45	–
TIGIT high *versus* low^e^	1.136 (0.519–2.513)	0.749	–
CD4 high *versus* low	1.57 (0.763–3.258)	0.222	–
CD8A high *versus* low	1.116 (0.54–2.317)	0.767	–
FOXP3 high *versus* low	0.891 (0.371–2.105)	0.793	–
CD16 high *versus* low	1.047 (0.505–2.176)	0.902	–
TOX high *versus* low	0.818 (0.381–1.754)	0.605	–

a One hundred twenty of 123 patients had fully evaluable clinical parameters and were included in the univariable and multivariable analyses.

b Low *versus* high ACE2 is as determined by *k*-mean clustering [Figure 1(a) and (b) for normal lung tissue and Supplemental Figure 1(a) and (b) for lung tumor tissue].

c The odds ratios were calculated using logistic regression (generalized linear model with binomial distribution) in R using the stats package.^24^ The odds ratios and Wald 95% CI were determined using the oddsratio in R.^25,26^ The univariable analysis indicated that TNM staging (1 *versus* ⩾1), PD-L1 (high *versus* low), ICOS (high *versus* low), CTLA-4 (high *versus* low), PD-1 (high *versus* low), and TIGIT (high *versus* low) were all significant by Wald test *p* value. These significant covariates were then used in the multivariable logistic model, in which TNM staging and ICOS were indicated significant.

d Of 64 patients with TNM stage >1 disease, 40 had high ACE2 expression; of 56 patients with TNM stage 1, 27 had high ACE2 expression in normal lung tissue.

e Low *versus* high expressors are as determined in Figure 1, panels D through K.

f Only covariates with *p* values < 0.05 in the univariable analysis were included in the multivariable analysis.

ACE2, angiotensin-converting enzyme 2; CI, confidence interval; ISOS, inducible T-cell co-stimulator; NSCLC, non-small cell lung cancer; TNM, tumor, nodes, and metastases.

**Figure 2.**
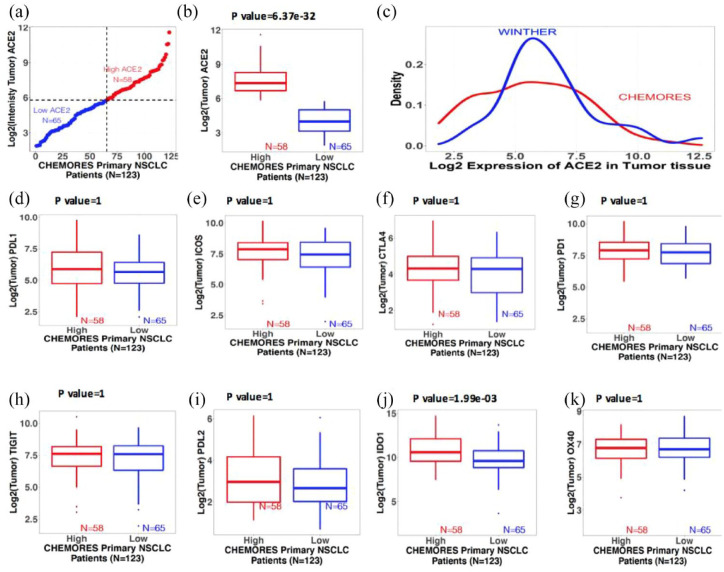
Trends of gene expression in tumor tissue from resected and metastatic NSCLC. Boxplots show the median (reflected by the line that divides the box into two parts) and the interquartile range (shown by the box itself). The points shown outside the box are outlier expression values. The significant difference between the expression values of the two risk groups derived using a two-sided Student’s *t* test with 95% confidence interval (see also Figure 1 for normal lung tissue). (a) Distribution of expression of ACE2 in tumors of resected NSCLC patients. X-axis: 123 resected NSCLC patients (CHEMORES) ranked from lowest to highest ACE2 expression. Y-axis: Log 2 intensity of expression of ACE2 in tumor tissue classified in high *versus* low using *k*-mean clustering. Red is high (*n* = 58) and blue is low (*n* = 65). Expression threshold in Log 2 scale was found to be around 5.8. (b) Boxplots of intensity of mRNA expression of ACE2 in tumor tissues from resected NSCLC (*n* = 123). (c) Density plot of the mRNA expression of ACE2 in tumor tissue from resectable NSCLC (CHEMORES dataset *n* = 123) and from metastatic NSCLC (WINTHER dataset *n* = 32). X-axis: Log 2 mRNA expression of ACE2. Y-axis: Density (abundance) of expression values of ACE2. (d) to (k) Boxplots of intensity of expression of immune checkpoint genes in tumor tissues from resected NSCLC (*n* = 123) in each risk group (high *versus* low ACE2 expression by *k*-means clustering): (d) PD-L1, (e) ICOS, (f) CTLA-4, (g) PD-1, (h) TIGIT, (i) PD-L2, (j) IDO1, and (k) OX40. Significant differences between the expression values of the immune-related genes in the high and low ACE2 groups were tested using a two-sided Student’s *t* test. All *p* values were adjusted for multiple comparisons *via* the Bonferroni correction (by multiplying the alpha by the number of comparisons). Bonferroni-adjusted *p* value < 0.05 is significant. ACE2, angiotensin-converting enzyme 2; ICOS, inducible T-cell co-stimulator; NSCLC, non-small cell lung cancer.

In contrast to the findings in normal lung tissue, *ACE2* transcript level in tumor tissue did not correlate with stage of disease [Table 1, Figure 2(a) to (c)].

We also analyzed *ACE2* transcript expression in normal lung tissue derived from 32 patients with metastatic NSCLC who participated in the WINTHER clinical trial (Supplemental Table 2)^25^; as seen in Figure 1(c), their expression was further shifted to the right in the curve, consistent with higher *ACE2* expression levels in normal lung tissue from patients with metastatic NSCLC *versus* that from patients with resectable NSCLC.

In contrast to the findings in normal lung tissue, *ACE2* transcript level in tumor did not correlate with stage of disease [Table 1, Figure 2(a) to (c)].

### Immune checkpoint transcript expression and T-cell infiltrate pattern in normal lung tissues differ in high *versus* low *ACE2* expressors

We explored the two groups of normal tissues from resected NSCLC (normal tissues with low *versus* high *ACE2* expression) and found distinct immunological profiles. Specifically, boxplots show [Figure 1(d) to (k)] that *PD-L1, ICOS, CTLA-4, PD-1*, and *TIGIT* had significantly greater RNA expression when *ACE2* expression was high *versus* low (after Bonferroni adjustment for multiple comparisons), while *PD-L2, IDO1*, and *OX40* showed no significant differences in the high *versus* low *ACE2* cohorts.

In contrast to the findings in normal lung tissue from our NSCLC patients, when we examined tumor tissue in this cohort, *ACE2* expression did not correlate with levels of *PD-L1, ICOS, CTLA-4, PD-1*, and *TIGIT* (Table 1, Figure 2).

The pattern of infiltrating T cells also differs in normal lung tissue of high and low ACE2 expressors (Table 1). In univariate analysis, expression of *CD8A* (a marker of cytotoxic T lymphocytes) and *CD16* (NK cells) is significantly higher in the high *ACE2* expressors group. Expression of *TOX*, a marker of T-cell exhaustion, is also significantly higher in the high ACE2 group.

### In a multivariable analysis, high *TOX* and *CD16* RNA expression was independently associated with high *ACE2* RNA expression in normal lung

While the immunoregulatory genes, specifically *PD-L1, ICOS, CTLA-4, PD-1*, and *TIGIT*, and the markers of T cells infiltrate *CD8A, CD16*, and *TOX* all had significantly higher RNA expression in the presence of higher *ACE2* expression in an univariable analysis [Figure 1(d) to (k)], Table 1 shows that, when entered into a multivariable analysis, only TNM stage >*1 (p = 0.04) and high TOX (p = 0.02) expression* correlated independently with high *versus* low *ACE2* expression in normal lung tissue; High *CD8A* and high *ICOS* showed a trend for association with high *ACE2* (CD8A *p* = 0.06, ICOS *p* = 0.08 multivariable).

### High *versus* low *ACE2* RNA expression in either normal lung tissue or tumor tissue from resected NSCLC was not associated with DFS

**Figure 3.**
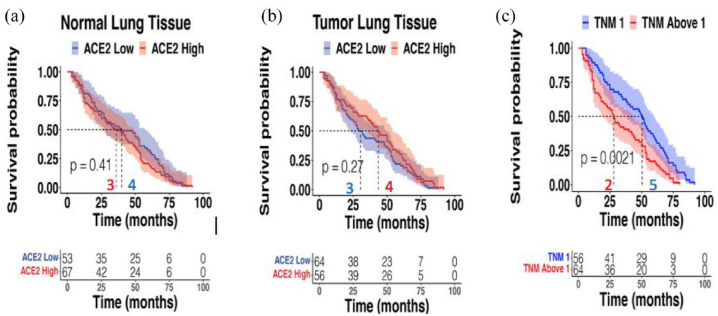
DFS correlation with ACE2 expression in normal or tumor lung tissue and TNM staging. CHEMORES dataset *n* = 120. Panel A shows no correlation between ACE2 expression in normal lung tissue of resected NSCLC and DFS [ACE2 expression dichotomized to high *versus* low as per data in Figure 1(a) and (b)]. Panel B shows no correlation between ACE2 transcript expression in tumor lung cancer tissue of resected NSCLC and DFS [ACE2 expression dichotomized to high *versus* low as per data in Figures 2(a) and (b)]. Panel C shows significant correlation between higher TNM staging and shorter DFS in resected NSCLC patients. ACE2, angiotensin-converting enzyme 2; DFS, disease-free survival; NSCLC, non-small cell lung cancer; TNM, tumor, nodes, and metastases.

We analyzed Kaplan–Meier DFS curves for high *versus* low *ACE2* expressors in both normal or tumor lung samples in the CHEMORES dataset of resected lung cancers and found no differences [Figure 3(a) and (b)]. Higher TNM staging, as expected, correlated with shorter DFS [Figure 3(c)].

## Discussion

Cancer patients are vulnerable to severe acute respiratory syndrome following coronavirus 2 (SARS-CoV-2: COVID-19) infection for many reasons including, but not limited to immunocompromise associated with cancer or cancer treatment, older age, comorbidities, and are more at risk due to repeated contacts with health-care facilities that may house COVID-19 patients. Furthermore, patients with lung cancer may be especially predisposed to COVID-19 disease, perhaps due to pulmonary compromise or other factors.^27–29^ By exploring normal lung tissues (in NSCLC patients not exposed to SARS-CoV-2 because the tissue collection predated the pandemic), we identified that the expression of *ACE2* in normal lung tissue or normal bronchial epithelium can distinguish two groups of patients with expected lower and higher vulnerability for serious COVID-19 pulmonary disease.^3^ This work was performed on two independent cohorts of patients: a cohort of 123 patients with primary resected NSCLC^20^ (246 paired distant normal and tumor tissues from surgery) and a cohort of 32 patients with metastatic NSCLC^21^ [64 paired matched analogous normal (bronchial mucosa) and tumor tissues biopsies]. High- and low-*ACE2* expression groups were discernible in both cohorts and in both normal and tumor tissues [Figures 1(a) and (b); Figures 2(a) and (b)]. Interestingly, more advanced stage of lung cancer disease correlated independently with higher *ACE2* expression levels in normal lung tissues [Figure 1(d) and Table 1]; this correlation with stage of disease was not seen for high *versus* low *ACE2* in tumor tissue [Table 1, Figure 2(c)]. High *ACE2* expression in itself did not however correlate with shorter DFS [Figure 3(a) and (b)] while, as expected, higher TNM stage did [Figure 3(c)]. The high- and low-expressing *ACE2* groups were, respectively, presumed at higher or lower risk of developing a severe form of COVID-19 following SARS-CoV-2 infection.^3,22,30^

Severe COVID-19 pulmonary infection may be at least partly ascribable to immune dysregulation.^31^ Similar to other respiratory viral infections, adaptive immune responses, especially of T cells, play an important part in SARS-CoV-2 infection. Even so, it remains a matter of debate whether T-cell responses are helpful or harmful in COVID-19, and whether these responses are dysfunctional, subpar, or excessive.^32^ Taken together, the literature suggests that patients afflicted with severe COVID-19 can have either insufficient or excessive T-cell responses.^32^ However, samples from lungs often have a low viral burden, suggesting that patients’ deaths may be attributable to uncontrolled host inflammatory processes rather than an active viral infection.^3,7–9,13^

Our data bring a novel insight into this debate. Analysis of specific markers of infiltrating T cells demonstrated that normal lung tissue with high ACE2 expression had a significantly higher infiltration with cytotoxic T lymphocytes (CD8A) and NKs (CD16) while the level of infiltrating T-regulatory and T-helper cells was not significantly different. However, this higher level of T-cell infiltrate is balanced by high expression of *TOX*, which is a marker of T-cell exhaustion, calling into question the functionality of the infiltrating immune cells^23^ and suggesting a mechanism of protection should antigen exposure produce an excessive activation and cellular response. These correlations have not been seen in tumor tissues suggesting a fundamental role of the normal lung tissue in the comorbidity of cancer and COVID-19.

Reminiscent of recent reports that examined lung autopsies from patients afflicted with severe COVID-19,^12^ we show significant RNA overexpression of *PD-1* and *CTLA-4* checkpoints in normal lung tissue of resected NSCLC patients [Figure 1(f) and (g)]; furthermore, *PD-1* and *CTLA-4* overexpression correlated with high *ACE2* levels (Table 1), the latter presumed to predispose to severe COVID-19 respiratory disease, probably because ACE2 is the entry receptor for COVID-19.^22,33^ PD-L1, the PD-1 ligand, is also overexpressed in the normal lung tissue of patients in this study when *ACE-2* levels are high [Figure 1(d) and Table 1]; finally, T-cell immunoglobulin and ITIM (immunoreceptor tyrosine based inhibitory motif) domain (TIGIT),^34^ another immune inhibitory receptor, is similarly overexpressed in our patients’ normal lung tissues harboring high *ACE2* transcripts [Figure 1(h) and Table 1]. Acquired cell-mediated immune defense T cells play a crucial role in clearing viral infections, thus reducing the severity of COVID-19’s symptoms. Our data suggest that an important feature of patients with COVID-19 may be T-lymphocyte exhaustion and impaired immune cell functionality. This is associated with expression of inhibitory immune checkpoints/ligands,^35^ consistent with the high levels of *PD-1, PD-L1, CTLA-4*, and *TIGIT* that we observed in high *ACE2* expressing normal lung tissues.

Intriguingly, however, while high levels of *PD-1, PD-L1, CTLA-4*, and *TIGIT* immune inhibitory transcripts in normal lung tissue all correlated with high normal lung *ACE2* transcripts in an univariate analysis, high *ICOS* expression was the only variable that showed a trend of correlation with high *ACE2* expression in multivariable analysis (*p* = 0.08) (Table 1). In contrast to the results of normal lung tissue, none of these factors was significantly correlated with high *ACE2* expression in tumor tissue.

ICOS (CD278) is an inducible co-stimulatory molecule for T-cell proliferation and cytokine secretion (including IL-4, IL-10, and IL-21, but IL-2 is inefficiently produced). It is the third member of the CD28 co-receptor family, which are all involved in regulating T-cell activation and adaptive immune responses. ICOS has significant homology with the other two family members co-stimulatory CD28 and co-inhibitory receptor CTLA-4,^18,36^ both of which are T-cell-specific cell surface receptors that regulate the immune system; CD28 potently promotes those T-cell activities that are crucial for an effective antigen-specific immune response; the homologous CTLA-4 offsets the CD28-mediated signals, and thus averts an otherwise potentially fatal lymphoid system overstimulation. ICOS, the third member of this family of molecules, matches CD28 in potency, and their roles in downstream signaling are similar but not identical;^37^ ICOS enhances T-cell foreign antigen responses, including proliferation, lymphokine secretion, and upregulation of molecules that promote cell–cell interaction. Although, IL-2 is not induced by ICOS, some cytokines, including IL-10 are super-induced. Together with its ligand (ICOSL), ICOS participates in the release of cytokines, stimulating immune response activation, and promotes T-cell activation and effector functions but, also, when sustained, inhibitory activities mediated by T-regulatory cells. In preclinical studies related to cancer, ICOS agonists potentiate the effects of anti-CTLA-4.^18^ Furthermore, ICOS is upregulated in the presence of anti-CTLA-4 treatment.^18^ In viral infection, ICOS is a marker of circulating T-follicular helper cells (cTfh); these cells induce viral-specific memory B cells to differentiate into plasma cells, whose levels correlate with protective antibody responses.^38^ Indeed, the emergence of ICOS-positive CD4+ T cells in the blood correlates with the development of protective antibody responses generated by memory B cells upon seasonal influenza vaccination .^37^

**Figure 4.**
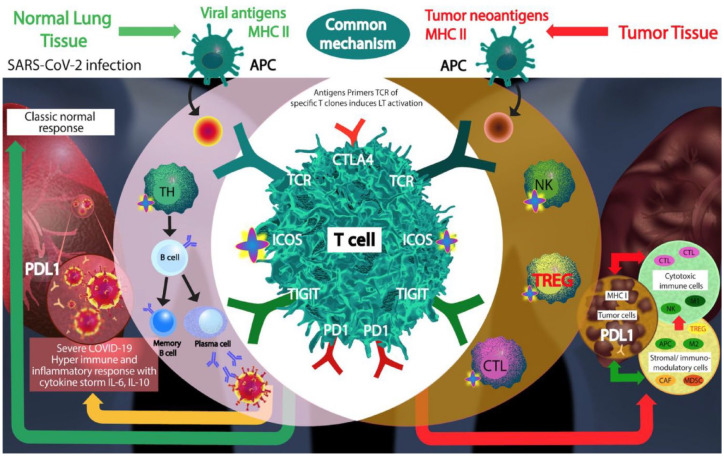
SARS-CoV-2 and cancer share similar mechanism of T-cell antigen-mediated immune response. Schematic representation of the COVID-19 and NSCLC comorbidity model. The clone of T cell that recognizes specifically the neo-antigen is activated, proliferates, and triggers onset of the cytotoxic (antitumor) and humoral immune response (neutralizing antibodies against SARS-CoV-2), globally coordinated by ICOS, which may also result in hyperinduction of cytokines. Immune checkpoints may dampen the antitumor and antiviral response. APC, professional antigen presenting cells; ICOS, inducible T-cell co-stimulator; M, macrophages; MHCII, major histocompatibility complex II; NK, natural killer cells; NSCLC, non-small cell lung cancer; T Cell, 457 LyTCD4+ that differentiates in TH, T helper, CTL, cytotoxic lymphocytes T (CD8+) restricted to CMH I; TREG, T-regulatory cells.

In conclusion, our work, expanding on prior reports,^5,10,39,40^ identified a similar mechanism through which SARS-CoV-2 and tumor cells may interact with the host immune system – the T-cell antigen-mediated immune response. Therefore, our transcriptomic observations may explain a potential comorbidity risk association between the two diseases (NSCLC and COVID-19 pneumonia) and could provide a rationale for new therapeutic strategies .^41,42^ We demonstrate that several different situations are at play in the normal lung tissues of NSCLC patients (Figure 4): (i) a higher prevalence of ACE2 receptors in individuals with more advanced stage lung disease, implying a greater risk of COVID-19 infection and severity of illness; (ii) a higher level of T cells infiltration but with a high expression of *TOX*, a marker of exhaustion suggesting impaired functionality of the infiltrating immune cells; and (iii) higher expression of PD-1, PD-L1, CTLA-4, and TIGIT immune inhibitory molecules, presumed to induce a strong immune blockade and an immune-tolerant profile, potentially relevant to susceptibility to both NSCLC and of COVID-19 illness. Importantly, however, expression of *ICOS*, an immune and cytokine stimulatory molecule and significantly higher (in univariate analysis) in high *ACE2*-expressing normal lung tissues from NSCLC patients. High levels of ICOS may explain the predisposition to an inflammatory/cytokine over-reaction in patients with higher *ACE2* expression, which may in turn underlie the more serious manifestations of COVID-19 pneumonia in individuals with NSCLC. These observations are compatible with prior work demonstrating infiltration with T cell and NK cells to be particularly pronounced in ACE2-high tumors.^39^ Therefore, subgroups of patients with NSCLC may have gene expression profiles in their normal lung tissue that promote heightened susceptibility to COVID-19 infection and a hyperimmune response. Intriguingly, these correlations between levels of ACE2 transcripts and immune regulatory molecules were not discerned in the tumor tissue.

This study presents several limitations: (i) high ACE2 levels are considered a surrogate for susceptibility to COVID-19 infection, and therefore further work would need to be performed in patients who later did or did not become infected; (ii) we could not determine whether ACE2 expression is higher in the normal lung of lung cancer patients than in individuals without lung cancer and whether such a difference might be the reason why patients are vulnerable to COVID-19; and (iii) patients with resectable and metastatic NSCLC are from different studies. Normal lung tissues were obtained differently (distant normal lung from surgically resected specimens in CHEMORES *versus* normal bronchial mucosa obtained by bronchoscopy in WINTHER). Thus, differences in ACE2 expression may not represent metastatic *versus* localized NSCLC, but alveolar *versus* bronchial tissues. There are also significant differences between patient populations in CHEMORES *versus* WINTHER trials (sex, smoking status, histology), which may also explain differences in ACE2 expression between the groups.

However, several observations argue in favor of the hypothesis associating high expression of ACE2 in normal lung tissues cancer and COVID-19 comorbidity: (i) higher levels of *ACE2* expression have been seen in lung tissues of patients without lung cancer, with severe COVID-19.^3^ (ii) In our study, in the primary resected NSCLC cohort, ACE2 expression is higher in patients with more advanced TNM as compared to stage 1; and (iii) the function of T-cell antigen-mediated response has a complex regulation, suggesting a balance between T-cell exhaustion and increased negative blockade and a high expression of ICOS.

Taken together, our findings suggest the importance of transcriptomic interrogation of normal lung tissue for unraveling the immune mechanisms that are key to the biology of COVID-19 infection and of lung cancer and need further investigation and validation in a prospective cohort of patients.

## Supplemental Material

sj-docx-1-tam-10.1177_17588359221133893 – Supplemental material for Comorbidity between lung cancer and COVID-19 pneumonia: role of immunoregulatory gene transcripts in high ACE2-expressing normal lung Click here for additional data file. Supplemental material, sj-docx-1-tam-10.1177_17588359221133893 for Comorbidity between lung cancer and COVID-19 pneumonia: role of immunoregulatory gene transcripts in high ACE2-expressing normal lung by Vladimir Lazar, Jacques Raynaud, Shai Magidi, Catherine Bresson, Jean-François Martini, Susan Galbraith, Fanny Wunder, Amir Onn, Gerald Batist, Nicolas Girard, Ulrik Lassen, C. S. Pramesh, Amal Al-Omari, Sadakatsu Ikeda, Guy Berchem, Jean-Yves Blay, Benjamin Solomon, Enriqueta Felip, Josep Tabernero, Eitan Rubin, Thierry Philip, Angel Porgador, Ioana Berindan-Neagoe, Richard L. Schilsky and Razelle Kurzrock in Therapeutic Advances in Medical Oncology

## Declarations

**Ethics approval and consent to participate:** The biobanking study CHEMORES was approved by the Institut Mutualiste Montsouris’s Ethics Committee. The collection of biopsies in WINTHER trial was approved by Ethics committee of each participating center.

**Consent for publication:** Ethics committees approved the use of research data for publication, without disclosure of any information related to identity of patients

**Availability of data and materials:** The data related to this article have been submitted to the Array Express data repository at the European Bioinformatics Institute (http://www.ebi.ac.uk/arrayexpress/) under the accession numbers E-MTAB-1132 (Array express – http://www.ebi.ac.uk/arrayexpress/) under the accession numbers E-MTAB-1132 (GE).^28^ For review process, all manuscript related data, related manuscript documents and the proprietary R codes are available at: https://drive.google.com/drive/folders/11NddvYJuGPfnYyx_QpbypwouBOnvnxxR?usp=sharing Codes are available upon request to corresponding author.
