# Supplementary material for: Comorbidity between lung cancer and COVID-19 pneumonia: role of immunoregulatory gene transcripts in high ACE2-expressing normal lung
Source: Ther Adv Med Oncol. 2022 Oct 28;14:17588359221133893. doi: 10.1177/17588359221133893 (PMC9618916; doi:10.1177/17588359221133893)
Supplement: sj-docx-1-tam-10.1177_17588359221133893 – Supplemental material for Comorbidity between lung cancer and COVID-19 pneumonia: role of immunoregulatory gene transcripts in high ACE2-expressing normal lung [file sj-docx-1-tam-10.1177_17588359221133893.docx]

**SUPPLEMENTARY DATA**

**Supplemental Table 1. Patient characteristics in CHEMORES dataset (n=123 patients with resectable NSCLC) [20]**

| CHEMORES NSCLC | n=123 (100%) |
| --- | --- |
| Age median (range) | 63 (40.9-84.6) |
| Males n (%) | 89 (72%) |
| Smoking Current | 64 (52%) |
| Former | 51 (42%) |
| Never | 8 (6%) |
| Histology AC | 57 (46%) |
| SCC | 50 (41%) |
| LCC | 13 (11%) |
| Other | 3 (2%) |
| Stage I | 56 (46%) |
| II | 27 (22%) |
| III  IV  Not determined | 32 (26%)  5 (4%)  3 (2%) |

***Abbreviations:*** *AC= adenocarcinoma; LCC= large cell carcinoma; SCC= squamous cell carcinom*

**Supplemental Table 2. Patient characteristics in WINTHER dataset (n=32 patients with metastatic NSCLC) [25]**

| WINTHER NSCLC | n=32 (100%) |
| --- | --- |
| Age median (range) | 60.5 (26-80) |
| Males n (%) | 18 (56%) |
| Smoking Current | 3 (9%) |
| Former | 8 (25%) |
| Never | 21 (66%) |
| Histology AC | 27 (85%) |
| SCC | 2 (6%) |
| LCC | 2 (6%) |
| Other | 1 (3%) |
